# Rhythm increases perceptual tolerance when measuring electrically evoked compound action potentials in cochlear implant recipients

**DOI:** 10.3389/fnins.2025.1711456

**Published:** 2026-01-05

**Authors:** Lutz Gärtner, Konrad Schwarz, Timo Bräcker, Wiebke Lamping, Thomas Lenarz, Andreas Büchner

**Affiliations:** 1 Department of Otolaryngology, Hannover Medical School, Hanover, Germany; 2 Research and Development, MED-EL Medical Electronics, Innsbruck, Austria; 3 MED-EL Research Center, MED-EL Elektromedizinische Geräte GmbH, Hanover, Germany

**Keywords:** auditory looming, cochlear implant, electrically evoked compound action potential, fine-grain, loudness perception

## Abstract

In cochlear implant (CI) recipients, measurement of the electrically evoked compound action potential (ECAP) is used in clinical routine to prove the electrode-nerve interface and to support fitting, especially in very young children. To record an ECAP amplitude growth function (AGF), electrical stimuli are presented at increasing intensities up to the maximum acceptable loudness (MAL). However, a continuously monotonically rising sound can quickly be perceived as unpleasant and thus impair the success rate of ECAP threshold determination. The present study investigates whether perceptual and objective parameters, which are involved in ECAP measurements, depend on the type of stimulus presentation. In 27 subjects with a CI (28 implants), stimuli of five different AGF patterns were presented ten times in random order at a medial electrode contact, resulting in a total of 50 AGFs per implant. One pattern (P_*clin*_) in which the stimuli increase strictly monotonically is already implemented in the clinical software (MAESTRO). The present study also employed rhythmic patterns and patterns with pauses. The behavioral threshold (THR), the maximum comfortable level (MCL), the MAL, and objective ECAP threshold were analyzed. ECAP threshold values did not change significantly when different stimulus paradigms were applied. Rhythmic AGF patterns were perceived as less unpleasant and enabled a higher MAL compared to P_*clin*_. This in turn led to improved ECAP threshold determination, with the success rate rising to 86.7%, compared to 79.1% for P_*clin*_.

## 1 Introduction

A cochlear implant (CI) is a medical device that restores hearing to individuals with severe to profound sensorineural hearing loss. The CI electrode array is surgically implanted in the scala tympani of the cochlea. The patient usually perceives an auditory sensation when neural tissue is activated by sufficient stimulus intensity. However, measuring the evoked action potentials requires a response from multiple nerve fibers. This response is referred to as an electrically evoked compound action potential (ECAP), and the patient often perceives a clearly audible to loud sound.

ECAP measurement in CI users has become a valuable objective method (He et al., 2017). Among many other applications, it enables intra- and postoperative verification of auditory nerve functionality, can help in detecting electrode migration (Gärtner et al., 2014) and device failure (Gärtner et al., 2016; Gärtner et al., 2021a), and supports speech processor fitting (Gärtner et al., 2022). CI manufacturers have integrated ECAP measurement capabilities into their systems. No additional equipment is needed beyond what is already required for speech processor fitting, making it easier to use in daily clinical practice.

There are several ways to estimate the ECAP threshold. Visual inspection of ECAP recordings at different stimulus levels by an experienced clinician seems to be the best solution. However, this is time-consuming and results vary depending on the examiner. Automated detection would therefore be preferable. In fact, many methods are already available (Botros et al., 2007). They all have in common that the stimulus strength increases over time during the ECAP recording. If the associated increase in loudness becomes too uncomfortable for the patient, ECAP threshold determination could fail.

The abbreviation TECAP is used differently in various publications. In the following, TECAP stands for ECAP threshold. In this study, we investigate the influence of different stimulus patterns on behavioral and objective parameters in CI users for the first time. It is known that in normal hearing subjects, rising sounds are perceived as more salient than receding sounds. This perceptual bias for looming sounds (Neuhoff, 1998) is attributed to the biological significance of survival: a continuously rising sound may be perceived as an impending threat. The brain may have evolved a bias or increased sensitivity to rising sounds in order to gain an advantage in escaping from predators. Therefore, the continuously monotonically rising tone during the recording of the ECAP amplitude growth function (AGF) can quickly be perceived as uncomfortable and thus lead to the termination of the measurement. However, the algorithm used for TECAP determination is based on sufficiently strong stimulus responses (see Materials and methods section). The results of this study show how these can be achieved without causing discomfort to the CI user.

## 2 Materials and methods

### 2.1 Ethics statement

The Ethics Committee of the Hannover Medical School, Germany, where the data were collected using the research software, approved the study (No. 6586). All participants gave written informed consent before any study-specific procedures were performed. All participants also gave written informed consent for the publication of the respective data.

### 2.2 Participants

This study comprises 28 cochlear implants (ID01 to ID28) in 27 participants (ID12 and ID17 belong to the same patient). Demographic data are presented in Table 1. Further data on etiology and audiological background are listed in Supplementary Table 1. All measurements were conducted between August 2018 and August 2019. Only adults were included in this study. Another inclusion criterion was that a CI with an i100 platform manufactured by MED-EL Medical Electronics (Innsbruck, Austria) had to be in use for compatibility with the ECAP measurement hardware and software. Users were asked to agree to participate in the study during their regular follow-up visit. There was no additional criterion for selection of participants. No additional visit was required for measurement. Participants were not compensated for their participation, and no travel expenses were paid.

**TABLE 1:** Participant demographics.

Implant ID	Gender	Age at implantation (years)	Duration of hearing with CI at time of measurement (years)	Implant type	Electrode type	Implanted side
ID01	M	68.7	5.3	Synchrony	Flex 28	R
ID02	M	38.7	0.7	Synchrony	Flex 28	L
ID03	M	40.7	5.1	Concerto	Flex 28	L
ID04	F	49.0	0.4	Synchrony	Flex 28	R
ID05	F	46.3	3.2	Synchrony	Flex 20	R
ID06	M	72.4	0.7	Synchrony	Flex 24	R
ID07	M	52.8	0.4	Synchrony	Flex 28	R
ID08	F	55.5	1.1	Synchrony	Flex 24	R
ID09	F	44.5	3.1	Synchrony	Flex 28	R
ID10	M	48.8	4.0	Synchrony	Flex 28	R
ID11	F	56.0	3.0	Synchrony	Flex 20	L
ID12	M	63.2	1.2	Synchrony	Flex 28	R
ID13	M	35.5	8.5	Sonata	Standard	L
ID14	F	63.7	6.1	Concerto	Standard	L
ID15	F	69.6	0.8	Synchrony	Flex 28	R
ID16	F	49.9	0.1	Synchrony	Flex 28	L
ID17	M	64.3	0.1	Synchrony	Flex 28	L
ID18	F	27.2	0.1	Synchrony	Flex 28	R
ID19	F	66.4	2.9	Synchrony	Flex 28	L
ID20	F	19.5	13.8	Pulsar	Standard	L
ID21	M	38.8	7.3	Concerto	Standard	L
ID22	F	21.1	0.8	Synchrony	Flex 28	R
ID23	F	50.5	2.7	Synchrony	Flex 16	L
ID24	M	45.9	2.3	Synchrony	Flex 28	L
ID25	M	63.3	1.4	Synchrony	Flex 24	L
ID26	M	47.1	1.1	Synchrony	Flex 24	R
ID27	F	58.2	1.1	Synchrony	Flex 28	R
ID28	M	58.0	0.6	Synchrony	Flex 28	L

M, male; F, female; R, right; L, left. Further audiological background including etiology, onset and duration of hearing loss, pre-implant hearing experience is provided by Supplementary Table 1. The length of the electrode array is as follows: Flex 16: 16 mm, Flex 20: 24 mm, Flex 28: 28 mm, Standard: 31.5 mm.

### 2.3 Stimuli and ECAP-Recordings

All measurements were conducted using the MAX USB interface box (MED-EL Company, Innsbruck, Austria) with the measurement coil placed over the implant. This system was driven by a personal computer running a research software. Bi-phasic, charge-balanced stimuli were presented to the medial electrode contact E06. Pulse duration was 40 μs (exceptions: 50 μs for ID20 and 60 μs for ID25), interphase gap 2.1 μs, and repetition rate 60 pulses per second. The measurement window captured responses up to 1.7 ms after stimulus onset. To reduce stimulus artifact, pulses were presented in pairs with alternating polarity and the response was subsequently averaged. A zero-amplitude template subtraction algorithm was applied to all recordings to compensate for the implant signature artifact. Four adjacent electrode contacts (E04, E05, E07, and E08) were used to record the response. Therefore, stimuli at a given stimulus strength were presented as alternating pairs four times in a row (0.13 s duration per level). The stimulus level was increased in 174 steps from 0 to 875 cu (where 1 current unit, cu, is equal to 1 μA), corresponding to a maximum charge of 35 nC.

For the first 11 implants, ID01–ID11, measurements were stored with reduced resolution only, and for the remaining 17 implants, ID12–ID28, the storage procedure was revised by making use of an adaptive Sigma-Delta modulation (Zierhofer, 2008) and ECAP measurements were stored identically to the clinical software MAESTRO, version 7 and higher (MED-EL Company, Innsbruck, Austria). For subsequent analysis, ECAP parameterization based on ECAP amplitudes showed no differences, nevertheless the signal-to-noise ratio of single traces was affected by the reduced resolution. This was evaluated for the 17 implants, ID12–ID28, by artificially reducing the data quality and comparing the results: correlation analysis using linear mixed models (LMM) and subject as random effect revealed a correlation of *r* = 0.984 between TECAPs calculated on the basis of the two storage qualities (see Supplementary Table 2).

The hardware setup and data processing are similar to those of the clinical setup and the clinical software, MAESTRO version 7 and higher (MED-EL Company, Innsbruck, Austria). The custom-made research software used enabled different ways to raise the stimulus level compared to the clinical software.

**FIGURE 1:**
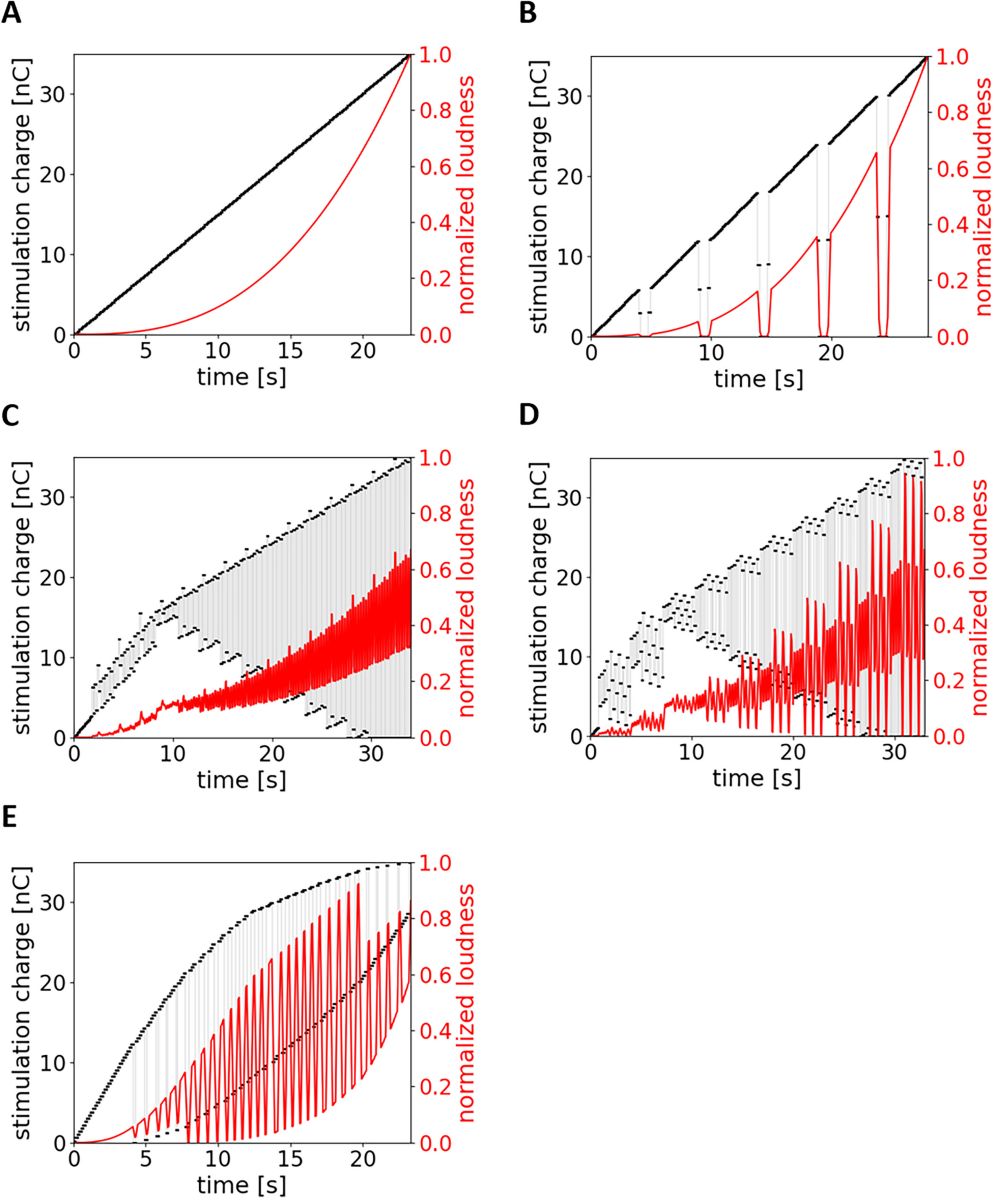
Different stimulus patterns for ECAP AGF recordings. **(A)** Strictly monotonic increasing, as is the current standard in clinical routine (P_*clin*_). **(B)** Monotonically increasing interspersed with pauses (P_1_). **(C)** Stimulus oscillates with a less pronounced rhythm (P_2_). **(D)** Stimulus oscillates in a more pronounced rhythm (P_3_). **(E)** Stimulus oscillates with pauses whose duration depends on the stimulus strength (P_4_). A sound sample for each pattern is available in Supplementary material.

Five different patterns of raising up the stimulus level were under investigation (Figure 1): the Fine-grain paradigm (Gärtner et al., 2018), as implemented in the clinical software MAESTRO (version 7 and higher), presents stimuli with a continuously increasing level (pattern P_*clin*_) (Figure 1A). As a variation, pauses of 1 s duration were added (pattern P_1_) (Figure 1B). In addition, three non-monotonic patterns were introduced. Pattern P_2_ (Figure 1C) had a light and P_3_ (Figure 1D) a stronger rhythm. To achieve this, pulses were rearranged within a set of 24 stimulus levels (i.e., a duration of 3.2 s). In the last paradigm, stimulus strength was periodically reduced with increasing duration (pattern P_4_) (Figure 1E), i.e., longer pauses (stimulus reduction) for stronger stimuli. A similar constant growth in stimulus intensity was present throughout the patterns in order to minimize bias for algorithms included in ECAP parameterization.

In Figure 1, the black dots and lines refer to the stimulus strength E in nC. The red lines refer to the theoretically perceived loudness L and were calculated using following equation posed by Fu and Shannon (1998):


$$\text{L}=\text{k}\,{\text{E}}^{q}$$

where *q* = 2.72. k is a constant factor. As loudness integration window of 200 μs was used.

An AGF was presented 10 times with each of the 5 paradigms (P_*clin*_, P_1_, P_2_, P_3_, P_4_). The order was pseudorandomized. This resulted in a total of 50 AGFs per implant ID. Sound samples of these five pattern are available as Supplementary material. The study was single-blinded. Therefore, only the conductor of the measurements knew which pattern was currently being tested.

### 2.4 ECAP parameterization

For all AGF patterns post-processing including filtering of ECAP traces, determination of N- and P-peaks of the ECAP response, parameterization of ECAP AGF with a sigmoid function, as well as selection of the most representative recording electrode was done using the AutoART algorithms (Strahl et al., 2018), as they are implemented in the clinical Software MAESTRO (version 7 and higher).

After recordings of a single run out of 50 for each implant, ECAP recordings for each stimulation pattern were sorted according to stimulus levels, separately for each recording electrode. The four resulting data sets were processed independently to derive four ECAP parameterizations. For each data set, ECAP amplitude at a given stimulus level was calculated using a moving average of ± 3 levels (7 values in total). ECAP amplitude [y(x)] as a function of stimulus level (x) was parameterized by fitting of a sigmoidal function


$$\text{y}\,\left(x\right)={\text{y}}_{0}+\frac{\text{c}}{1+{\text{e}}^{-k\,\left(x-x_{0}\right)}}$$

using the Levenberg-Marquardt algorithm (Marquardt, 1963). Here, *y*_0_ (noise level, i.e. the spontaneous activity of the neuron without electrical stimulation, in μV), *c* (the maximum observable ECAP amplitude at a given recording electrode, in μV), *x*_0_ (stimulus strength, in nC, corresponding to the inflection point of a symmetric sigmoidal function), and *k* (representing the rate of change per nC within the dynamic range of the modeled neuronal population) are the parameters to be fitted (van de Heyning et al., 2016), which were then used to estimate TECAP and the slope of the ECAP AGF at the steepest point of the sigmoid. The latter is defined by the value of the first order derivative at *x*_0_:


$$\text{s}\,l\,o\,p\,e={\text{y}}^{{}^{\prime }}\,\left({\text{x}}_{0}\right)=\frac{\text{c}\cdot \text{k}\cdot {\text{e}}^{-k\,\left(x-x_{0}\right)}}{{\left(1+{\text{e}}^{-k\,\left(x-x_{0}\right)}\right)}^{2}}=\frac{\text{c}\cdot \text{k}}{4}$$

The stimulation intensity at which the slope intersects with y_0_ was used to denote TECAP:


$$\text{T}ECAP={\text{x}}_{0}-\frac{\text{y}\,\left({\text{x}}_{0}\right)-{\text{y}}_{0}}{\text{s}\,l\,o\,p\,e}={\text{x}}_{0}-\left(\left({\text{y}}_{0}+\frac{\text{c}}{1+{\text{e}}^{-k\,\left(x-x_{0}\right)}}\right)-{\text{y}}_{0}\right)/$$

$$\frac{\text{c}\cdot \text{k}}{4}={\text{x}}_{0}-\frac{2}{\text{k}}$$

From the four parameterizations derived in this way (one for each recording contact), the AutoART algorithms were also used to decide whether a TECAP value could be estimated (i.e., whether the quality of the approximation to a sigmoidal function was sufficient) and which recording electrode provided the best result. Therefore, the final data set for each stimulation electrode contains one recording electrode. If no TECAP could be reliably determined, the remaining objective parameters (y_0_, x_0_, c, k, ECAP slope) were also not included in the statistical evaluations.

### 2.5 Behavioral loudness perception tests

The ECAP AGF measurements were accompanied by behavioral perception tests. Subjects were instructed to press the space bar of the personal computer three times during the presentation of each AGF. The first response indicates the threshold level (THR), i.e., when sound becomes audible. The second response indicates the most comfortable level (MCL), i.e., when sound reaches a pleasantly loud perception. If sound becomes uncomfortably loud, a third response indicates the maximum acceptable loudness (MAL). Due to the limited maximum presentation level of 35 nC, reaching the MAL was not necessarily expected. At the end of the experiment, participants were asked to rate which pattern sounded most pleasant to them. Therefore, the ranking was given as a subjective response, where the subjects could replay representative 5 s for each paradigm.

### 2.6 Variability and intrinsic parameters of test methods

Loudness levels, such as threshold, are best identified using stepwise approaches that bracket the level of interest (Carhart and Jerger, 1959). Repeated short stimuli avoid effects such as loudness masking, and stepwise approaches facilitate the identification of different loudness levels. The present experiment, however, aims at identifying and quantifying these influencing factors such as looming (continuous, not stepwise increase of stimulus levels) and loudness masking. Ten repetitions for each paradigm were chosen as an a priori compromise between accuracy and duration of the experiment.

To estimate the variability of the determined parameters (THR, MCL, MAL, TECAP, ECAP slope, y_0_), the maximum, minimum, and mean values were first determined for each parameter in each of the 10 runs per paradigm. The variability is then calculated as the median across all 28 implants of the difference between the maximum and minimum values, divided by the mean value [(max-min)/mean].

### 2.7 Statistical analysis

Python programming software (version 3.6, 2017), was used to program the research tool and store databases using besides built-in functionality packages numpy (version 1.13), PyQt5 (version 5.10.1, 2018), sqlite3 (version 3.23, 2018), and the “Research Interface Box 2” dynamic link library RIB2.dll (version 1.71, provided by the University of Innsbruck) to stimulate with the CI via MAX Interface Box (Litovsky et al., 2017).

For statistical analyses, python (version 3.7.3, 2019) with updated packages of the research tool and in addition matplotlib (version 3.1.0, 2019) and scipy (version 1.7.1, 2021) were used. Via jupyter notebook (version 6.4.8, 2021) and the package rpy2 (version 3.4.5, 2021) the “R programming language and statistical environment” was used to perform statistical analyses (R Core Team, 2021)(R Core Team, 2021), version 4.1.1, with packages “lmerTest” (Kuznetsova et al., 2017), “lme4” (Bates et al., 2015), “MuMin” (Burnham and Anderson, 2004), and “performance” (Nakagawa et al., 2017), function “lmer” and “r2” for the LMM, “multcomp” (Hothorn et al., 2008), and functions “glht” and “squaredGLMM” for the *post-hoc* analysis.

For subsequent statistical comparisons in this study, LMMs using fixed and random factors were used. The details of the used factors are given in the appropriate section.

## 3 Results

### 3.1 General

The average duration of a complete study session was 43 min 5 s per subject (22 min 44 s—69 min 9 s). Occasionally, the subjects took a break, which averaged 2 min 16 s (8 s—7 min 24 s).

In 8 out of 1,400 cases (0.57%), no perceptual thresholds (THR, MCL, and MAL) could be determined because no data was saved due to an error. Similarly, in 71 out of 1,400 cases (5.07%), no objective measures could be determined (i.e., values for y_0_, c, k, x_0_, TECAP, and ECAP slope), again due to an error resulting in no data being saved.

The level of MCL in particular, but also THR and MAL, is chosen individually by each subject, so that comparisons of absolute levels between subjects are not valid. Here we are comparing between different stimulus patterns per subject.

In order to comply with the loudness safety guidelines of the ethics committee, a stimulation limit of 35 nC was set. A ceiling effect was considered to have occurred if a perceptual threshold was above 34 nC. Several subjects reached this limit (see Table 2), and it must be taken into account in the following statistics.

**TABLE 2:** Variability of behavioral limits and frequency of ceiling effects for each of the five stimulus patterns.

Parameter	P_*clin*_	P_1_	P_2_	P_3_	P_4_
THR variability	19.2%	17.7%	14.3%	16.1%	21.1%
THR ceiling frequency	0	0	0	0	0
MCL variability	25.5%	25.2%	25.7%	24.4%	22.8%
MCL ceiling frequency	2 (m: 2, f: 0)	2 (m: 2, f: 0)	2 (m: 2, f: 0)	3 (m: 2, f: 1)	4 (m: 3, f: 1)
MAL variability	29.4%	26.8%	26.8%	29.1%	18.5%
MAL ceiling frequency	13 (m: 8, f: 5)	14 (m: 9, f: 5)	21 (m: 11, f: 10)	22 (m: 11, f: 11)	20 (m: 11, f: 9)

The variability of threshold (THR), maximum comfort level (MCL), and maximum acceptable loudness (MAL) is expressed as median across implants of (max-min)/mean for each of the five stimulus paradigms (P_*clin*_, P_1_, P_2_, P_3_, P_4_). If a behavioral limit exceeded a limit of 34 nC in more than 3 out of 10 runs, a ceiling effect was considered. The frequency of such ceiling effects is specified according to gender (m, male; f, female).

Besides averaged values, 10 repetitions as a test-retest scenario provide insights. Over the duration of the experiment, y_0_ showed a high variation (see Table 3). The algorithms work as expected in terms of TECAP accuracy as well as for ECAP slope accuracy, with the latter exhibiting greater variation. This is due to the fact that the selected parameter set is chosen among several recording electrodes. While the TECAP is less affected by the recording electrodes, the ECAP amplitude, and, consequently the ECAP slope change with distance, i.e., with the specific location of the stimulation and recording electrodes. For the evaluation of differences between the paradigms, the factor recording electrode is taken into account.

**TABLE 3:** Variability of ECAP parametrization and success rate in TECAP determination for each of the five stimulus patterns.

Parameter	P_*clin*_	P_1_	P_2_	P_3_	P_4_
TECAP variability	16.8%	14.0%	14.7%	15.9%	13.0%
ECAP slope variability	45.8%	41.1%	40.1%	39.7%	33.7%
y_0_ variability	169.0%	123.7%	149.3%	196.0%	179.5%
Success rate	79.1%	77.9%	82.2%	84.4%	86.7%
Effective number of runs	259	265	270	265	270

The variability of TECAP, ECAP slope, and y_0_ is expressed as median across implants of (max-min)/mean for each of the five stimulus paradigms (P_*clin*_, P_1_, P_2_, P_3_, P_4_). In very few cases, a series of measured values could not be saved, meaning that the effective number of runs was < 280.

Overall, the ECAP parameterization in this study was found to be robust in a test-retest scenario with a TECAP variability of 16.8% for the clinically implemented pattern P_*clin*_.

### 3.2 ECAP measurements

The TECAP determination success rate was defined as the number of TECAP values determined divided by the number of runs (usually 10). For safety reasons, any unexpected event was handled in abortion of the current run and starting with the next one. In those instances, the attainment of 10 runs was impeded by data loss that occurred with the research software. The success rate was 79% for P_*clin*_, and for the new AGF stimulus patterns 78% (P_1_), 82% (P_2_), 84% (P_3_), and 87% (P_4_). More details are shown in Table 3.

**FIGURE 2:**
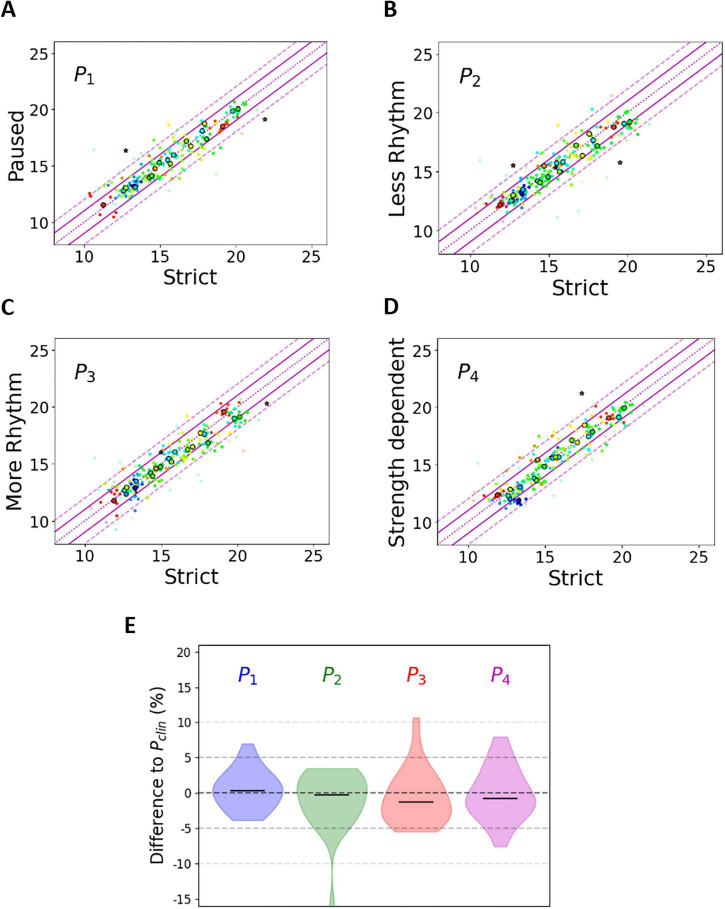
Effect of stimulus pattern on TECAP. **(A–D)** The *x*-axis shows TECAP values (nC) determined by using the strict stimulus pattern P_*clin*_. The *y*-axis shows TECAP values (nC) determined by using the newly introduced stimulus patterns P_1_, P_2_, P_3_, P_4_. Individual measurements (up to 10 per paradigm) are shown as colored dots (28 colors for 28 implants) without borders. The corresponding mean values per implant are shown as colored dots with black borders if a TECAP value could be determined in more than 3 cases, otherwise as stars with black borders. Lilac lines are shown to guide the eye. **(E)** Overview of data already shown in **(A–D)** as difference to pattern P_*clin*_.

The influence of stimulus pattern on estimated TECAP is shown in Figure 2. Figures 2A–D show the TECAP of the P_*clin*_ pattern on the *x*-axis, while the TECAP of the P_1_, P_2_, P_3_, and P_4_ patterns are shown on the *y*-axis. Figure 2E shows the difference in TECAP of the 4 newly designed paradigms compared to the classic strict Fine-grain pattern P_*clin*_. Correlation analysis revealed similar TECAP for all paradigms with Pearson’s correlation coefficients (vs. P_*clin*_) = 0.899 (P_1_), 0.881 (P_2_), 0.885 (P_3_), and 0.908 (P_4_). Pairwise comparisons did not reveal any statistical difference of TECAP between P_*clin*_ and any of the other stimulus patterns.

#### 3.2.1 Linear mixed model

We performed LMM analysis as explained in Statistical Analysis. Investigations of ECAP parameter (TECAP, ECAP slope, y_0_) were performed using the model


ECAP⁢parameter∼paradigm+(1|subject)+

(1|recording⁢_⁢electrode).

Estimates of the effects and significance are shown in Table 4.

**TABLE 4:** Linear mixed model analysis of objective ECAP parameters TECAP, ECAP slope, and y_0_.

Parameter	P_1_	P_2_	P_3_	P_4_
TECAP (nC)	+0.0	–0.1	–0.2	–0.1
ECAP slope (μV/nC)	–0.7	+0.5	+1.0	+0.5
y_0_ (μV)	–1.3	–1.0	+1.7	+3.2

“ECAP parameter∼ paradigm + (1| subject) + (1| recording_electrode)” revealed no significant differences (*p* < 0.05) for tested parameters, except for: TECAP, ECAP slope for “P_3_” resp. y_0_ for “P_4_.”

In contrast to single pairwise comparisons, TECAP and ECAP slope showed a significant difference for paradigm P_3_ (*p* < 0.05) and y_0_ for paradigm P_4_ (*p* = < 0.05).

### 3.3 Behavioral loudness perception tests

In very few of the 1,400 runs were the perception levels reported by the test subjects clearly incorrect. This concerned three runs in which THR was reported as zero (ID11, one run with P_2_; ID23, one run each with P_1_ and P_3_). Three other runs in which THR was below 3 nC could be discussed, as this value appears too low (ID 23, one run each with P_1_ and P_*clin*_; ID26, one run with P_2_). In one of these subjects (ID23), MCL and MAL were reported with values below 3 nC, which was highly unlikely (one run each with P_1_, P_3_, and P_*clin*_). In one case (ID13, one run with pattern P3), the feedback at THR was apparently forgotten, as THR, MCL, and MAL were identical (34.77 nC) and THR was significantly lower in the other runs. Nevertheless, the data from each test subject was included in the evaluation as provided by the test subject without correction.

**FIGURE 3:**
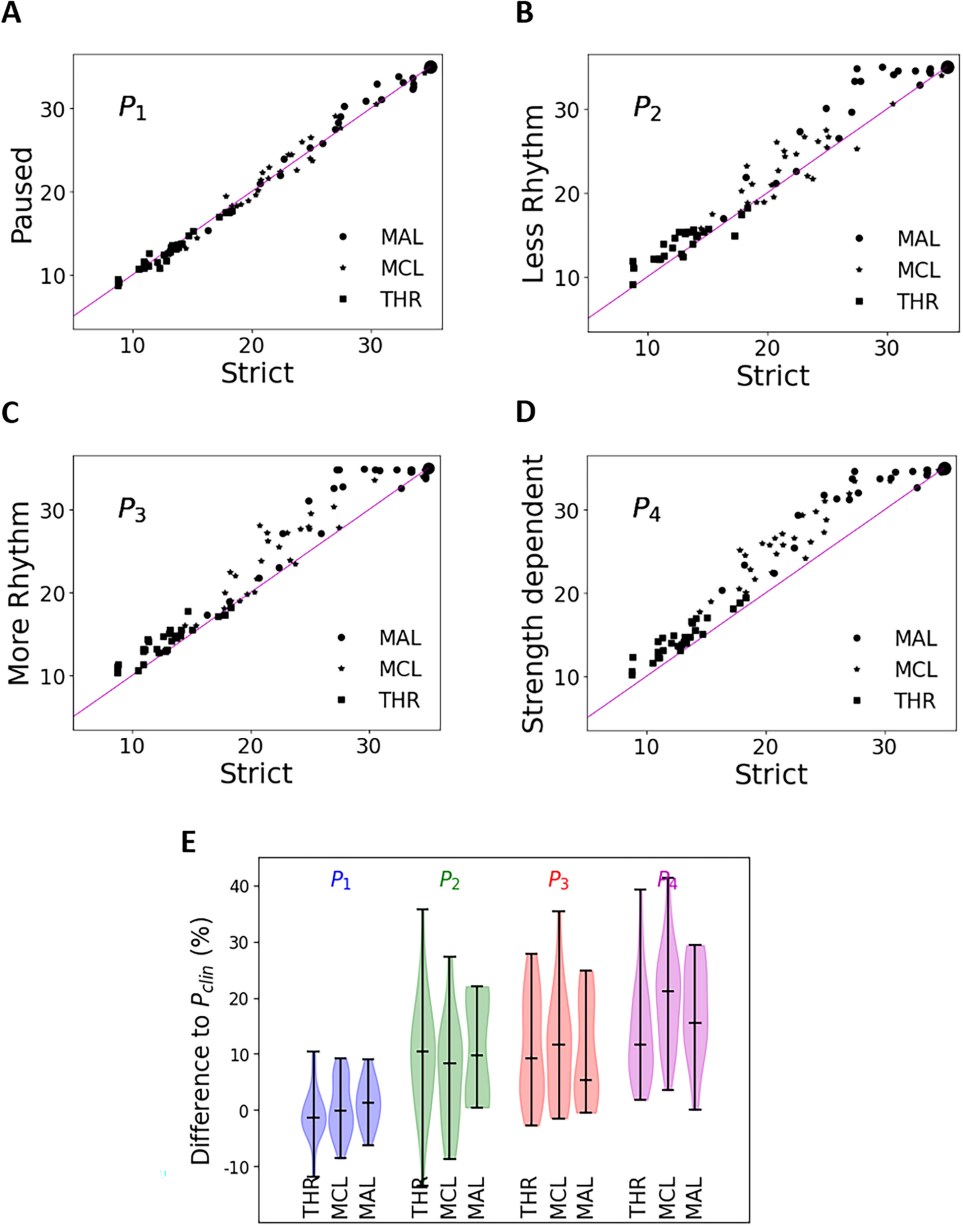
Effect of stimulus pattern on loudness perception. **(A–D)** The *x*-axis shows the stimulus charge (nC) of behavioral limits [threshold (THR), maximum comfort level (MCL), and maximum acceptable loudness (MAL)] for the strict P_*clin*_ pattern. The *y*-axis shows the stimulus charge (nC) of the corresponding limits for the newly introduced AGF paradigms. The lilac line indicates the same stimulus strengths for orientation. **(E)** Overview of data already shown in **(A–D)** as difference to pattern P_*clin*_.

The results of the behavioral tests are shown in Figure 3. In Figures 3A–D, values of stimulus charge for THR, MCL, and MAL values are indicated by different symbols. The size of the symbol reflects the amount of data with the same value. The *x*-axis shows the stimulus charge of the P_*clin*_ paradigm. The *y*-axis shows the stimulus charge of the newly introduced AGF paradigms. The relative difference compared to the P_*clin*_ pattern is shown in Figure 3E. The differences become more pronounced with increasing stimulus strength. As expected, our motivated AGF patterns had an effect on loudness perception. All data are available in Supplementary Table 3.

**FIGURE 4:**
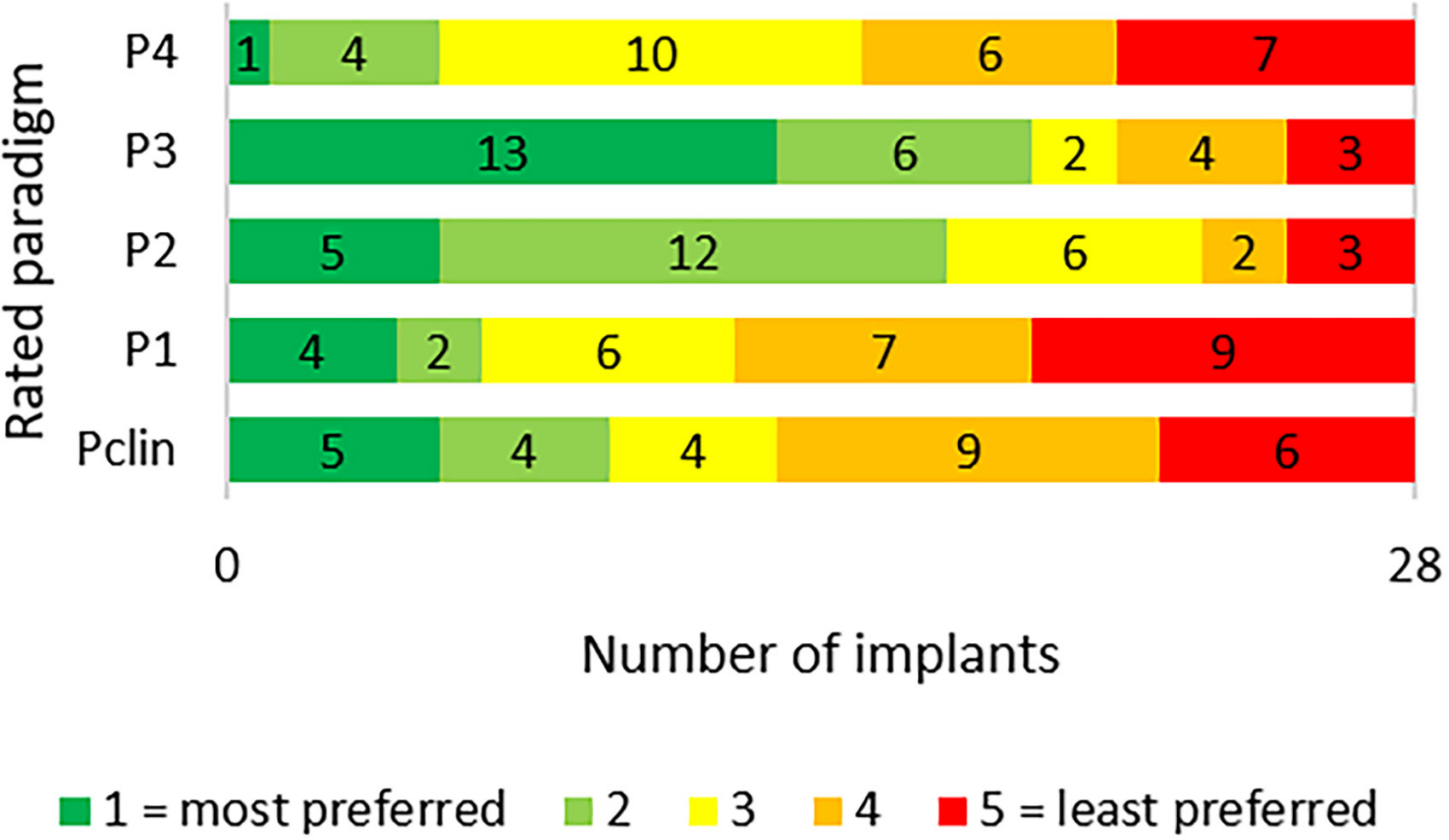
Subjective preferences regarding the various stimulus paradigms. The graph shows how often each pattern was rated as the most preferred (1) to the least preferred (5).

Results of subjective preferences are shown in Figure 4. Paradigms with rhythm P_2_ and P_3_ were rated highest. Interestingly, the bilateral patient with implant ID12 and ID17 ranked both ears different. Since he was the only candidate who participated in the study with both of his CIs, one can only speculate about the causes. ID12 was measured first, followed by ID17 2 days later. Different preferences could have been due to daily moods. The different periods of use/acclimatization could also have had an influence. ID17 was implanted 1 year after ID 12 (see Table 1). The preference did not correlate with TECAP determination success rate.

#### 3.3.1 Linear mixed model

We performed LMM analysis as explained in Statistical Analysis. Investigations of behavioral parameter (THR, MCL, and MAL) were performed using the model


behavioral⁢parameter∼paradigm+(1|subject)

once with all data, and once using only data below ceiling limit. Estimates of the effects and significance are shown in Table 5.

**TABLE 5:** Linear mixed model analysis of behavioral parameters THR, MCL, and MAL.

Parameter	P_1_	P_2_	P_3_	P_4_
THR (nC)	–0.2 (–0.2)	+1.1 (+1.1)	+1.3 (+1.3)	+1.7 (+1.7)
MCL (nC)	–0.0 (+0.2)	+1.0 (+1.1)	+2.1 (+2.2)	+4.0 (+3.9)
MAL (nC)	+0.1 (0.2)	+1.8 (+1.8)	+1.4 (+1.9)	+3.7 (+2.4)

“Behavioral parameter∼ paradigm + (1| subject)” revealed significant differences (*p* < 0.001) for all three parameters and paradigms P_2_, P_3_, and P_4_, but for no parameter P_*clin*_ vs. P_1_. Limiting data with respect to ceiling showed similar results. Data without ceiling effect in parentheses.

The effect of gender was examined using the LMM behavioral


parameter∼gender+(1|paradigm)

and revealed a significant effect (*p* < 0.001) for all behavioral parameters of 1.6 nC for MAL (2.7 nC including ceiled data), 2.2 nC for MCL (3.5 nC including ceiled data), and 1.1 nC (1.1 nC including ceiled data) for THR. Pairwise tests between female and male groups separated by paradigms did not reveal statistical significance.

## 4 Discussion

The algorithm implemented in the MAESTRO clinical software (version 7 and higher) for determining TECAP uses an approximation of the recorded AGF data to a sigmoidal function. The accuracy increases with increasing stimulus intensity. However, the patient then also perceives signals that are continuously getting louder. In acoustic hearing, a perceptual bias for rising sound is already known (Neuhoff, 1998). Sounds that increase in intensity are perceived as louder and change in loudness more than sounds that decrease in intensity. The effect is more pronounced for tones than for noise (Neuhoff, 2001).

If a perceptual bias for rising tones is also present in CI users, measuring the ECAP AGF with strict up-ramp stimulus intensities (P_*clin*_) may not be the best solution. The novelty of this study lies in the introduction of alternative AGF patterns by adding pauses and rhythm in an attempt to avoid continuous auditory looming, which should ultimately make the measurement more comfortable. The focus of this study was not to find out, whether a perceptual bias is also present in CI users, but to improve the ECAP measurement procedure and TECAP determination. Loudness integration of strict up-ramp stimuli may be reduced by masking effects: soft pulses following strong pulses cannot be heard and are perceived as pauses. Rearranging soft pulses after louder ones thus creates a rhythm and is hypothesized to be more comfortable to the user.

One limitation of this study is that only four patterns were tested in addition to the classic strict Fine-grain AGF pattern P_*clin*_. There is still room for further studies addressing different AGF patterns.

The rationale for the four patterns presented in this study was to reduce a potential perceptual bias for looming sounds and to make overall impression more comfortable for the subjects. Paradigm P_1_ could not reduce the perceived loudness as much as the other test paradigms (difference at MCL < 0.5 nC), so either the pauses used were too few to inhibit auditory looming, respectively change loudness perception, or the looming effect is not dominant below MAL levels in paradigm P_*clin*_. Paradigm P_4_, many pauses with stimulus-dependent duration, showed a strong effect (differences P_4_ – P_*clin*_ ∼ 4 nC, which is ∼20%) (see Figure 3E). We conclude that the method used to avoid looming, respectively, loudness masking, has a great potential. Although P_4_ had the largest loudness reduction, users did not rate it as pleasant (see Figure 4). Pattern P_2_ was rated as moderately pleasant and pattern P_3_ as most pleasant, suggesting that users recognize the intended rhythm and prefer stimulation with rhythmic patterns. Since the difference in loudness integration is only minor, auditory looming is more inhibited by the stronger rhythm, i.e., deviation from monotonic increment is greater. Our results show increased MCLs for both paradigms, but the difference P_3_ – P_*clin*_ = 2.1 nC is greater than P_2_ – P_*clin*_ = 1.0 nC. For MAL, ceiling effects bias the results: both paradigms increase MAL to a large extend. From this perspective, we conclude that loudness integration is a stronger effect than auditory looming.

Neuhoff and Heckel (2004) reported that looming has gender-specific effects. They found that as intensity increased, females perceived more loudness change than males, which is consistent with a more pronounced awareness of danger. In our study, we also find gender effects, but we were not able to quantify differences in this effect for individual paradigms with our data and experimental design.

The overall loudness of ECAP recordings can be influenced by different ways of estimating TECAP. Approximating a sigmoid function may require a higher stimulus level. However, there are other ways of estimating TECAP, for example by comparing signals in different time windows (Hoth et al., 2018), which has recently been made applicable to Fine-grain recordings (Gärtner et al., 2021b). We see effects on ECAP recordings for paradigm P_4_ on y_0_, which we connect to a larger number of recordings for subthreshold stimulation recorded at different times during the AGF. We also link these additional recordings to the improved test-retest accuracy for pattern P_4_ (TECAP variability: median across implants of (max-min)/mean < 13%), which was best among all paradigms. The differences for pattern P_3_ for TECAP and ECAP slope cannot be directly explained by this additional source of noise. In the future, other rhythmic patterns could be examined to investigate possible systematic effects, such as the frequency of changes between strong and soft pulses.

The Fine-grain method (P_*clin*_) has already led to a 5% higher success rate in determining TECAP compared to the former standard ART method (Garrido et al., 2018). The present study shows that TECAP estimation can be further improved by skillfully re-sorting the stimuli, i.e., by introducing rhythm. The use of different non-monotonous AFG patterns may contribute to further improvement.

## 5 Conclusion

In CI recipients, a monotonically increasing stimulus intensity during ECAP recordings can be perceived as unpleasant. Our study shows that optimized loudness integration increases overall achievable levels, but not comfort. Rhythmic stimuli that deviate more from the monotonic increment show higher attainable levels as well as greater pleasantness. This suggests that in addition to loudness integration, other effects such as the hypothesized looming effect play a role in ECAP recordings. Loudness integration has been observed to have a strong influence on perceived loudness even at very low stimulation rates, such as those used for ECAP recordings. ECAP measurements may provide more comfort when rhythm is introduced without altering the TECAP measurement results.

Sounds that continuously increase in intensity, such as those perceived during ECAP AGF measurement, may compromise successful TECAP determination. A better alternative is to use rhythmic stimuli, which also sound more comfortable to most subjects. The underlying effect may be attributed to a perceptual bias for rising sounds and/or loudness integration. TECAP values did not change significantly while applying different stimulus paradigms.

## Data Availability

The original contributions presented in this study are included in this article/Supplementary material, further inquiries can be directed to the corresponding author.
