# Supplementary material for: Rhythm increases perceptual tolerance when measuring electrically evoked compound action potentials in cochlear implant recipients
Source: Front Neurosci. 2026 Jan 5;19:1711456. doi: 10.3389/fnins.2025.1711456 (PMC12813165; doi:10.3389/fnins.2025.1711456)
Supplement: Supplementary file 2 [file Table_2.docx]

## Supplementary Material 1

## Comparison of reduced data quality vs. revised data quality

For the first ten subjects, data was stored only in a reduced data quality. The difference resulted from usage respectively not usage of an adaptive Sigma-Delta modulation to convert analog input into digital zeros and ones. For the first ten subjects, the signal was converted using a non-adaptive conversion, which resulted in a reduced signal resolution. This enabled the software to compress recorded data to about 10% of the size compared to using an adaptive conversion as well as faster process speed. However, a revision of the tool enabled storing a higher quality of data with sufficient speed.

The evaluation was performed in the second 17 subjects by artificially remodeling the stored data to reduced quality and comparing the results. This was done using a linear mixed model

threshold_low_quality~threshold_high_quality + (1|subject)

with resulting correlation coefficients for the two calculation variants of r^2=0.975.
